# QuantiMus: A Machine Learning-Based Approach for High Precision Analysis of Skeletal Muscle Morphology

**DOI:** 10.3389/fphys.2019.01416

**Published:** 2019-11-29

**Authors:** Jenna M. Kastenschmidt, Kyle L. Ellefsen, Ali H. Mannaa, Jesse J. Giebel, Rayan Yahia, Rachel E. Ayer, Phillip Pham, Rodolfo Rios, Sylvia A. Vetrone, Tahseen Mozaffar, S. Armando Villalta

**Affiliations:** ^1^Department of Physiology and Biophysics, University of California, Irvine, Irvine, CA, United States; ^2^Institute for Immunology, University of California, Irvine, Irvine, CA, United States; ^3^Department of Neurobiology and Behavior, University of California, Irvine, Irvine, CA, United States; ^4^Department of Biology, Whittier College, Whittier, CA, United States; ^5^Department of Neurology, University of California, Irvine, Irvine, CA, United States; ^6^Department of Orthopaedic Surgery, University of California, Irvine, Irvine, CA, United States; ^7^Department of Pathology and Laboratory Medicine, University of California, Irvine, Irvine, CA, United States

**Keywords:** muscle regeneration, cross-sectional area, central nucleation, Duchenne muscular dystrophy, machine learning, histological analysis, myofiber typing, mdx

## Abstract

Skeletal muscle injury provokes a regenerative response, characterized by the *de novo* generation of myofibers that are distinguished by central nucleation and re-expression of developmentally restricted genes. In addition to these characteristics, myofiber cross-sectional area (CSA) is widely used to evaluate muscle hypertrophic and regenerative responses. Here, we introduce QuantiMus, a free software program that uses machine learning algorithms to quantify muscle morphology and molecular features with high precision and quick processing-time. The ability of QuantiMus to define and measure myofibers was compared to manual measurement or other automated software programs. QuantiMus rapidly and accurately defined total myofibers and measured CSA with comparable performance but quantified the CSA of centrally-nucleated fibers (CNFs) with greater precision compared to other software. It additionally quantified the fluorescence intensity of individual myofibers of human and mouse muscle, which was used to assess the distribution of myofiber type, based on the myosin heavy chain isoform that was expressed. Furthermore, analysis of entire quadriceps cross-sections of healthy and mdx mice showed that dystrophic muscle had an increased frequency of Evans blue dye^+^ injured myofibers. QuantiMus also revealed that the proportion of centrally nucleated, regenerating myofibers that express embryonic myosin heavy chain (eMyHC) or neural cell adhesion molecule (NCAM) were increased in dystrophic mice. Our findings reveal that QuantiMus has several advantages over existing software. The unique self-learning capacity of the machine learning algorithms provides superior accuracy and the ability to rapidly interrogate the complete muscle section. These qualities increase rigor and reproducibility by avoiding methods that rely on the sampling of representative areas of a section. This is of particular importance for the analysis of dystrophic muscle given the “patchy” distribution of muscle pathology. QuantiMus is an open source tool, allowing customization to meet investigator-specific needs and provides novel analytical approaches for quantifying muscle morphology.

## Introduction

Acute trauma, prolonged periods of mechanical unloading or genetic mutations can all independently cause skeletal muscle cell death, atrophy, and changes in myofiber cross-sectional area (CSA). The resilience of skeletal muscle to overcome these environmental and genetic insults is partly attributed to its highly adaptive and regenerative capacity (Dumont et al., 2015). Although mechanical load influences myofiber CSA, muscle injury and regeneration provoke a larger variance in CSA because of the *de novo* formation of growing myofibers (Torres and Duchen, 1987; McDonald et al., 2015). In addition to their variance in CSA, developing myofibers express embryonic myosin heavy chain (eMyHC) and neural cell adhesion molecule (NCAM) during regeneration (Schiaffino et al., 1986; Dubois et al., 1994; Capkovic et al., 2008; Rochlin et al., 2010; Tedesco et al., 2010; Dumont et al., 2015). Thus, measuring the frequency or expression of regeneration markers and CSA is frequently used to quantitatively assess muscle regeneration (Covault and Sanes, 1985; Schiaffino et al., 1986; Illa et al., 1992; Dubois et al., 1994; Charlton et al., 2000; Capkovic et al., 2008).

The manual quantification of myofiber type, CSA, and centrally nucleated fibers (CNF) by histological methods is time-consuming and prone to user bias, negatively affecting the quality of data. Further, quantifying protein expression by microscopy methods is difficult because several factors (sensitivity and dynamic range of the imaging system; specificity of the antibodies; technical anomalies; inappropriately performing image post-processing prior to image analysis) can comprise the proportional relationship between protein expression and fluorescence intensity. Recently, multiple groups have successfully developed software that addresses the above limitations for the semi-automated, morphometric analysis of healthy skeletal muscle. For example, the Semi-automatic Muscle Analysis using Segmentation of Histology (SMASH) method was developed as an open source MATLAB application that measures myofiber properties such as size (CSA and minimum Feret diameter), CNFs, and myofiber type in immunofluorescence-labeled images (Smith and Barton, 2014). More recently, MyoVision was developed to evaluate CSA, myofiber type, and myonuclear number (Wen et al., 2017). Although MyoVision does not have the function to quantify CNFs, the software expands the automated potential of these aforementioned histological analyses by using algorithms that decrease the amount of user supervision.

Current software packages reliably assess the morphology of healthy muscle, in which morphometric features are uniform. However, the performance of a subset of these packages has not been validated in more complex model systems that vary greatly in morphology. Accurate assessment of diseased muscle [e.g., dystrophic muscle of the mdx mouse model of Duchenne muscular dystrophy (DMD)] is challenged by muscle necrosis and inflammation and constant tissue remodeling that contributes to large variance in myofiber size and increased interstitial tissue (Torres and Duchen, 1987; McDonald et al., 2015). We found that these complex disease features hinder the ability of existing software from discerning a true myofiber from artifact. To circumvent this limitation, we developed QuantiMus, a machine learning-based tool that uses a support vector machine (SVM) algorithm (Artan, 2011) to define myofibers with high fidelity. QuantiMus can be downloaded at https://quantimus.github.io. QuantiMus was developed as a plugin for the software program Flika (Ellefsen et al., 2014), which can be downloaded at https://flika-org.github.io.

QuantiMus integrates the analytical features of previous morphometric software programs, such as measurement of CSA and CNFs (Smith and Barton, 2014; Wen et al., 2017), with the capability to measure myofiber fluorescence intensity. Together, these features provide a single tool to simultaneously quantify fluorescence intensity, CNFs and CSA, and the use of machine learning algorithms reduces processing time and computing power. We compared the performance of QuantiMus to other semi-automated methods and validated that this tool accurately determined myofiber CSA and CNFs in healthy and diseased skeletal muscle. QuantiMus rapidly determined the proportion of type I and II myofibers in healthy mouse and human skeletal muscle, and the CSA of each myofiber type. QuantiMus also quantified the frequency of Evans blue dye (EBD), NCAM, or eMyHC positive myofibers in dystrophic muscle, and measured their fluorescence intensity. Collectively, we demonstrate the utility of QuantiMus as a tool for the rapid and rigorous quantification of multiple molecular and morphological features of skeletal muscle during homeostasis and disease.

## Materials

### Ethical Approval

Deidentified frozen, muscle cross-sections from archived human muscle biopsies were provided by UCI pathology laboratory, and their identity remained confidential throughout the study. Prior to biopsy collection, participants were informed about the requirements and potential risks of the procedures before providing their written informed consent. Biopsies were collected from patients because of a suspected inflammatory myopathy, which after pathological assessment revealed no skeletal muscle involvement. The experimental procedures adhered to the standards in the latest revision of the *Declaration of Helsinki* and were approved by the Institutional Review Board at the University of California, Irvine (HS#2016-3191).

### Animal Models

In compliance with the federal regulations, the use of mice in our study was approved by the University of California Irvine Institutional Animal Care and Use Committee. Mice were housed in a temperature-controlled facility under a standard 12 h light-12 h dark cycle with food and water provided *ad libitum*. C57BL/10 wildtype and C57BL/10ScSn-Dmd^mdx^/J (mdx) mice were originally obtained from Jackson laboratory (Bar Harbour, ME) and breeding colonies were maintained in-house. Mice were euthanized at 4 weeks of age with carbon dioxide using a gradual fill method per American Veterinary Medical Association guidelines, followed by cervical dislocation. For Evans blue dye (EBD) injected mice, animals were interperitoneally injected with a 1% EBD solution at a dose of 50 mg/kg, 16 h before euthanasia.

### Mouse Tissue Preparation

Quadriceps were isolated from 4-week-old WT and mdx mice. Quadriceps were embedded in optimal cutting temperature (O.C.T) compound (Sakaura Fine Tech, 25608-930), frozen in liquid nitrogen-cooled isopentane for 1 min and stored at −80°C. Eight-micron cross-sections were prepared on a Leica CM1950 cryostat, mounted on positively charged microscope slides, and stored at −80°C until the time of staining. Although most section thicknesses can be accommodated, we choose 8 μm as the optimal thickness for image quantification in this study; sections less than 8 μm resulted in a larger occurrence of gaps in laminin labeling, whereas sections greater than 8 μm yielded artifactual laminin labeling that obscured the myofiber perimeter.

### Immunofluorescence Labeling and Imaging

All immunofluorescent labeling procedures were performed with routine and validated protocols (Schiaffino et al., 1986; Illa et al., 1992; Capkovic et al., 2008; Wen et al., 2017). Sections were all labeled on the same day to eliminate inter-experimental variation. Images were acquired in a manner to ensure that fluorescence signals were not saturated, and specificity of the stain was ensured by comparison to control sections in which the primary antibody was omitted. Fluorescently labeled sections were protected from light through the staining procedure and image acquisition. Further, the measurement of fluorescence intensity was done on unaltered images (i.e., native brightness and contrast settings were never manipulated). A RGB image was converted to its single channel components and saved as an eight-bit TIFF file for compatibility with the QuantiMus pipeline.

#### Myofiber Regeneration and Laminin

To quantify the frequency and CSA of regenerating myofibers, frozen cross-sections of mouse quadriceps were labeled with anti-laminin, anti-NCAM, and anti-eMyHC antibodies (Table 1). Briefly, cross-sections were fixed with 2% paraformaldehyde for 5 min, and endogenous biotin was blocked with an avidin/biotin blocking kit (Vector Laboratories) per manufacturer instructions. Following washes with 1X PBS, endogenous mouse IgG was blocked with Mouse-on-Mouse blocking reagent (Vector laboratories) for 1 h at room temperature (RT). Muscle sections were washed in 1X PBS and incubated for 5 min at RT in blocking buffer comprised of 1X Tris-buffered saline with 2.5% normal donkey serum. Primary antibodies against laminin, NCAM, and eMyHC were diluted in blocking buffer at concentrations described in Table 1 and were incubated with sections for 1 h at RT. Primary antibodies were detected by indirect immunofluorescence staining with Alexa 488- and Alexa 647-conjugated secondary antibodies (Table 1) for 1 h at RT, protected from light. For the detection of eMyHC labeling, sections were incubated with biotinylated anti-mouse antibodies for 10 min followed by labeling with Alexa 594-conjugated streptavidin for 5 min. Sections were counter-stained with 4′,6-diamidino-2-phenylindole, dihydrochloride (DAPI, Sigma, 1.2 nM in 1X PBS) for 10 min to visualize nuclei. For the staining of laminin in human muscle, sections were fixed as described above and were blocked with 5% normal donkey serum, 3% BSA and 0.05% Tween-20 in 1X Tris-buffered saline for 1 h. Blocking solution was removed and sections were stained with anti-laminin antibody for 1 h at RT. Sections were washed and stained with secondary antibodies and DAPI as described above.

**Table 1:** Antibodies used for histology.

Antibody/labeling reagents	Vendor	Clone	Dilution	Final (μg/ml)
eMyHC	DSHB	F.1652	30	0.6
NCAM	SCBT	H28-123	200	0.5
Rabbit anti-laminin	Sigma	Polyclonal	200	2.5
Biotin anti-mouse IgG	Jackson Immuno	Polyclonal	80	15
MyHC type I	DSHB	BA-D5	200	1.4
MyHC type IIa	DSHB	SC-71	200	1.9
MyHC type IIb	DSHB	BF-F3	200	2
MyHC type IIx	DSHB	6H1	20	1.1
Streptavidin Alexa Fluor 594	Invitrogen	Polyclonal	80	25.0
Anti-Rat Alexa Flour 488	Invitrogen	Polyclonal	200	10
Anti-Rabbit Alexa Flour 647	Invitrogen	Polyclonal	200	10
Anti-Mouse IgG 2b DyLight 405	Jackson Immuno	Polyclonal	400	4.3
Anti-Mouse IgG1 Cy2	Jackson Immuno	Polyclonal	400	4
Anti-Mouse IgM DyLight 594	Jackson Immuno	Polyclonal	400/1500^a^	3.8/1

a *Final dilution of 1:400 was used for mouse sections. 1:1,500 was used for human*.

#### Myofiber Typing

The immunofluorescent detection of myofiber expression of myosin heavy chain isoforms was performed as previously described (Wen et al., 2017). Briefly, mouse quadriceps cross-sections were air-dried for 10 min, rehydrated with 1X PBS, and were blocked with Mouse-On-Mouse blocking reagent (Vector laboratories) for 1 h at RT. Cross-sections were stained with type I, IIa, IIb MyHC-specific antibodies, and anti-laminin (Table 1) for 1.5 h. For human samples, antibodies against myofiber type IIx were used instead of type IIb (Table 1). To detect myofiber types and laminin, sections were stained with secondary antibodies Dylight 405 goat anti-mouse IgG2b, Cy2 goat anti-mouse IgG1, Dylight 594 goat anti-mouse IgM, and anti-rabbit Alexa 647 (Table 1) to detect type I, IIa, IIb/x MyHC, and laminin staining, respectively. Sections were washed with 1X PBS and mounted for imaging. All tissue sections were imaged with a Keyence BZ-X700 inverted fluorescence microscope with a 10X (human) or 20X (mouse) objective and were stitched using BZ-X Analyzer software (Keyence).

### FIJI Analysis

To define muscle myofibers, images of laminin-stained cross-sections were first imported into FIJI and converted to binary images using the “make binary” function. The FIJI “wand tool” (legacy mode, tolerance = 0) was used to define the edge of operator-selected myofibers. The area (μm^2^) and minimum Feret diameter (μm) of these operator-selected myofibers were measured using the FIJI “measure” function. To manually define CNFs, DAPI-stained images were binarized and overlaid onto corresponding binarized images of laminin-stained sections. Operators used the FIJI “wand tool” (legacy mode, tolerance = 0) to manually select CNFs. The CSA of CNFs was measured using the FIJI “measure” function.

### Semi-automatic Muscle Analysis Using Segmentation of Histology Analysis

Because SMASH (version 5) requires RGB files for analysis, we used FIJI to convert acquired eight-bit greyscale images to a RGB format. As previously described (Smith and Barton, 2014), the detection of myofibers and CNFs using SMASH was performed using the following parameters: pixel size (μm/pixel) = 1.216 (mouse) or 1.780 (human); segmentation filter = 8 (mouse) or 25 (human); minimum fiber area = 12 (mouse) or 1,000 μm^2^ (human); and maximum fiber area = 5000 (mouse) or 20,000 μm^2^ (human). For the determination of CNFs, the following settings were used: distance from edge = 1.5 μm; nuclear size = 5 μm^2^; and nuclear smoothing = 5.

### MyoVision Analysis

Images of laminin-stained sections were analyzed using MyoVision Basic (version 1) as previously described (Wen et al., 2017). Various settings were tested, and the optimal following values were used: minimum area = 10; maximum area = 5,000 (mouse) or 50,000 (human); and pixel/μm = 1. Myofiber size was manually converted to micrometer after export.

### Determination of Accuracy and Variance

As previously described, accuracy for the number of defined myofibers, average CSA, and number of CNFs was determined by Eq. 1, where test software is defined as QuantiMus, MyoVision, or SMASH, and manual measurement are values obtained by FIJI analysis (Wen et al., 2017).

(1) Accuracy=1−TestSoftwareValue−ManualMeasurementManualMeasurement×100%

Coefficient of variation (CV) is defined by Eq. 2.

(2) $$CV=\left(\frac{\mathrm{Standard}\;\mathrm{Deviation}}{\mathrm{Average}}\right)\times 100$$

Data are expressed as the average ± standard error of the mean (SEM) or individual replicate values where indicated.

### Statistics

All statistical analyses were performed using GraphPad Prism Version 7.0 (GraphPad Software, Inc.). A two-way repeated measures ANOVA with a multiple comparison test (main column effect) was performed for fiber counting, CNF detection, and CSA accuracy measurements. A paired two-tailed *t*-test was performed for the analysis of the accuracy of CNF number and average area of CNFs. Statistical analysis for comparisons of WT and mdx measurements was performed using an unpaired two-tailed *t*-test with Welch’s correction.

## Methods

### Overview of the QuantiMus Pipeline

**Figure 1:**
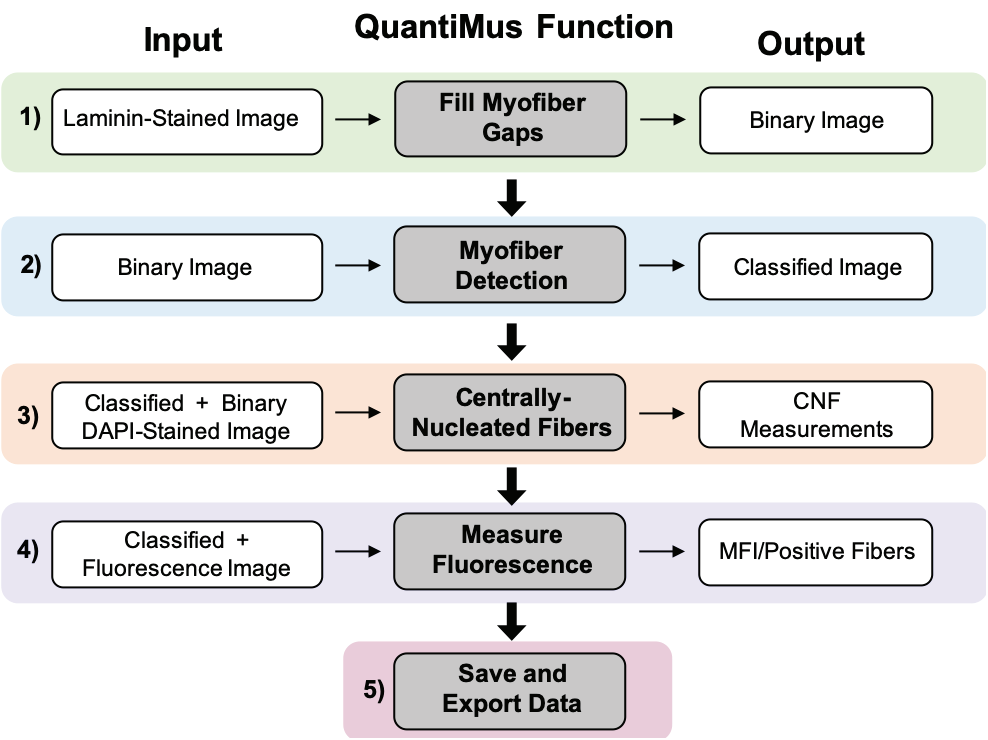
QuantiMus application workflow. The QuantiMus analysis pipeline is composed of five steps: (1) the “Fill Myofiber Gaps” function; (2) “Myofiber Detection” function; (3) “Centrally-Nucleated Fibers” function; (4) “Measure Fluorescence” function; and (5) “Save and Export Data” function.

The QuantiMus analysis pipeline is composed of five steps. Laminin-stained images of muscle cross-sections are first imported into the QuantiMus software. The “Fill Myofiber Gaps” function (Figure 1, Step 1) ensures accurate boundary definition by filling-in discontinuous regions of the laminin stain, which is used to define the myofiber perimeter and generate a binary image used in the myofiber detection function. The “Myofiber Detection” function accurately classifies regions of interest (ROI) as myofibers (Figure 1, Step 2), generating a classified image used in downstream functions. The “Centrally-Nucleated Fibers” function (Figure 1, Step 3) defines myofibers with centrally located nuclei by overlaying binarized images of laminin-corresponding DAPI-stained images onto the classified image. The “Measure Fluorescence” function of the QuantiMus pipeline (Figure 1, Step 4) allows users to measure myofiber fluorescence intensity emanating from immunofluorescence-labeled proteins. The “Save and Export Data” function is used to export processed data as an Excel sheet for further analysis and statistical testing (Figure 1, Step 5).

### Segmentation of Myofibers

Image segmentation assists the automation of image analysis by partitioning pixels of similar characteristics to define the boundary of an object (Liu and Structures, 2009; Zaitoun and Aqel, 2015). However, structural deformities in the tissue or technical artifacts in the staining procedure that result in discontinuous boundaries prevents current segmentation methods to accurately determine the myofiber perimeter (Kostrominova et al., 2013). To circumvent this limitation, we developed an algorithm to fill discontinuous myofiber boundaries (i.e., “gaps”) by coupling thresholding techniques with novel methods we developed and describe below.

**Figure 2:**
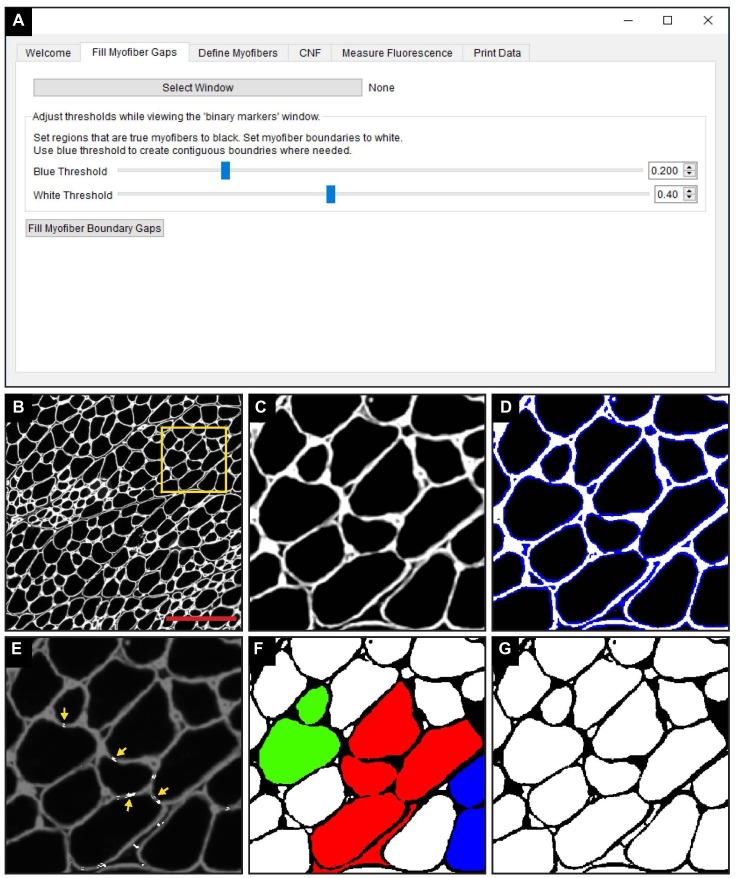
The “Fill Myofiber Gaps” function corrects gaps in myofiber boundaries that hinder single myofiber discrimination. **(A)** Screenshot of the QuantiMus user interface used for the “Fill Myofiber Gaps” function. **(B)** Representative image of a cross-section of 4-week-old mdx quadriceps, stained with anti-laminin antibody (white). **(C)** Zoomed in region of the cross-section in **(B)** (yellow box). **(D)** Interactive display showing thresholds set by the user with sliders in the Fill Myofiber Gaps Tab. **(E)** Myofiber gaps detected and filled by the algorithm (white regions highlighted by yellow arrows). **(F)** Binary image generated from the cross-section in **(C)** that was not corrected with the “Fill Myofiber Gaps” function; colored regions indicate grouped ROIs incorrectly detected as one myofiber. **(G)** Binary image generated from the cross-section in **(C)** that was corrected using the “Fill Myofiber Gaps” function. Scale bar = 200 μm.

The “Fill Myofiber Gaps” function is executed within the “Fill Myofiber Gaps” tab of the QuantiMus user interface (Figure 2A). TIFF images of laminin-stained sections (Figures 2B,C) are first imported and the user defines the myofiber boundary by manipulating the “Blue Threshold” and “White Threshold” sliders within the “Fill Myofiber Gaps” tab, such that the blue indicates the thickest possible boundary and white indicates the thinnest (Figures 2A,D). These slider settings are used to generate an evenly spaced list of thresholding values that are sequentially applied to the original image to form a series of binary images. Contiguous pixels from the binary image resulting from the lowest threshold value are grouped together into regions that become seeds for segmented regions. Each region of pixels is compared to the corresponding region from the binary image generated with the next higher threshold value. If the area of a respective region exceeds a defined size and increases by more than 20%, the region is considered to have exceeded the boundaries of the candidate myofiber and is erroneously “expanded.” In this case, a “white filler” (Figure 2E, yellow arrows) is inserted at the intersection of the original and “expanded” regions. These two steps – evaluating increases in candidate myofiber area and building borders when the increase is too large – are repeated for every region in each binary image of the series. Contiguous regions of pixels remaining at the end of this process are considered segmented regions and are fed into the next step of the QuantiMus pipeline. Binarization alone fails to resolve gaps within the myofiber perimeter, leading to the misclassification of adjacent myofibers as one combined region. An example of this is illustrated in Figure 2F, where each colored region demonstrates the incorrect clustering of multiple fibers. The “Fill Myofiber Gaps” methodology fills in breaks within the myofiber boundary to generate a binarized image (Figure 2G) with “corrected” myofiber detection. Although breaks in non-myofiber regions may also be filled in, these erroneously segmented regions are not classified as true myofibers during the “Myofiber detection” step (Figure 1 Step 2).

### Binary Classification of Myofibers and Interstitial Space

**Figure 3:**
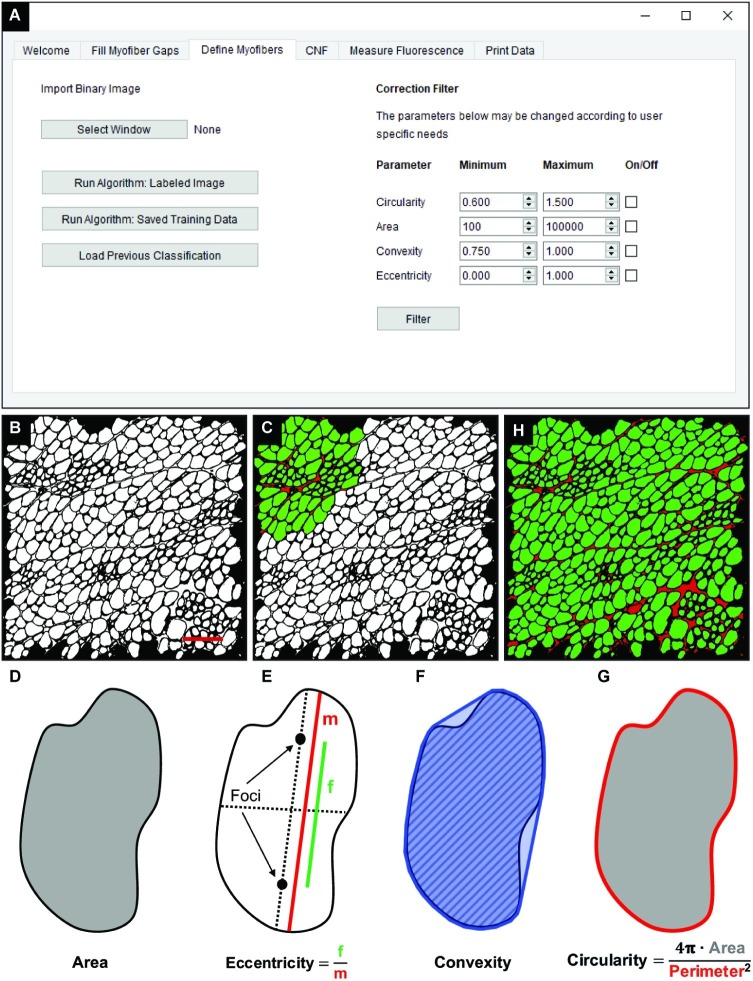
Classification of skeletal muscle myofibers. **(A)** Image of the QuantiMus user interface used to classify myofibers. **(B)** Binary image of quadriceps from 4-week-old mdx mice generated during the “Fill Myofiber Gaps” function. **(C)** Binary image highlighting user-classified ROIs (an ROI is defined as any contiguous region of pixels) used to train the machine learning algorithm for subsequent automated ROI classification. Green ROIs = Myofibers, red ROIs = interstitial space and artifacts. **(D–G)** Properties used to classify regions as myofibers. **(D)** Area (gray) is the total number of pixels in the region. **(E)** Eccentricity is calculated by dividing the focal distance (f, green line) by the major axis length (m, red line). Focal distance is defined as the length between the foci and the major axis length is the longest diameter of a region. **(F)** Convexity of an ROI is calculated by dividing the area (hatched area) by the convex area (blue area). The convex area is defined as the area within the smallest convex polygon that can be drawn around a region. **(G)** Circularity is determined using Eq. 3 as shown. Area = gray region, perimeter = red boundary. **(H)** A representative final image rendered by automated classification subsequently used for myofiber quantification and downstream analysis. Scale bar = 200 μm.

The “Fill Myofiber Gaps” function yields a binary segmented image that contains contiguous regions of pixels that are converted to unique regions of interest (ROIs) in the “Define Myofibers” tab (Figure 3A). We used a machine learning algorithm, a support vector machine (SVM), to accurately classify ROIs as myofibers. Initially, binary images are selected (Figure 3B) in the “Define Myofibers” tab, and the user manually classifies ROIs into two categories. As shown in Figure 3C, user-selected green ROIs are categorized as myofibers, whereas red ROIs are categorized as non-myofiber features (i.e., interstitial space or artifact). Four properties are determined for each ROI using functions from the open source scikit-image toolbox: area, eccentricity, convexity, and circularity (Van Der Walt et al., 2014). Area is defined as the number of pixels within each ROI (Figure 3D, gray area). Eccentricity describes the ellipticity of a region and is defined as the focal distance (Figure 3E, green line) divided by the major axis length (Figure 3E, red line). Convexity is defined as the ratio between the area (Figure 3F, hatched area) and the convex area of a region (Figure 3F, blue area) which is the area of the smallest convex polygon that encloses the region. Circularity (Figure 3G) describes the roundness of a region and is calculated as in Eq. 3.

(3) $$\mathrm{Circularity}=\frac{4\pi \cdot \mathrm{Area}}{{\mathrm{Perimeter}}^{2}}$$

These properties are then used to train an SVM with a radial bias function as the kernel, which is implemented from the open source scikit-learn library for machine learning in Python (Pedregosa et al., 2012). The trained SVM can then be applied to classify ROIs of the remaining image (Figure 3H). Training data can also be saved and later used to classify ROIs in binarized images of multiple muscle samples, reducing analysis time. Furthermore, the “Correction Filter” feature within the “Define Myofibers” tab provides a degree of flexibility that allows users to remove incorrectly classified myofibers (Figure 3A).

### Defining Centrally Nucleated Fibers

**Figure 4:**
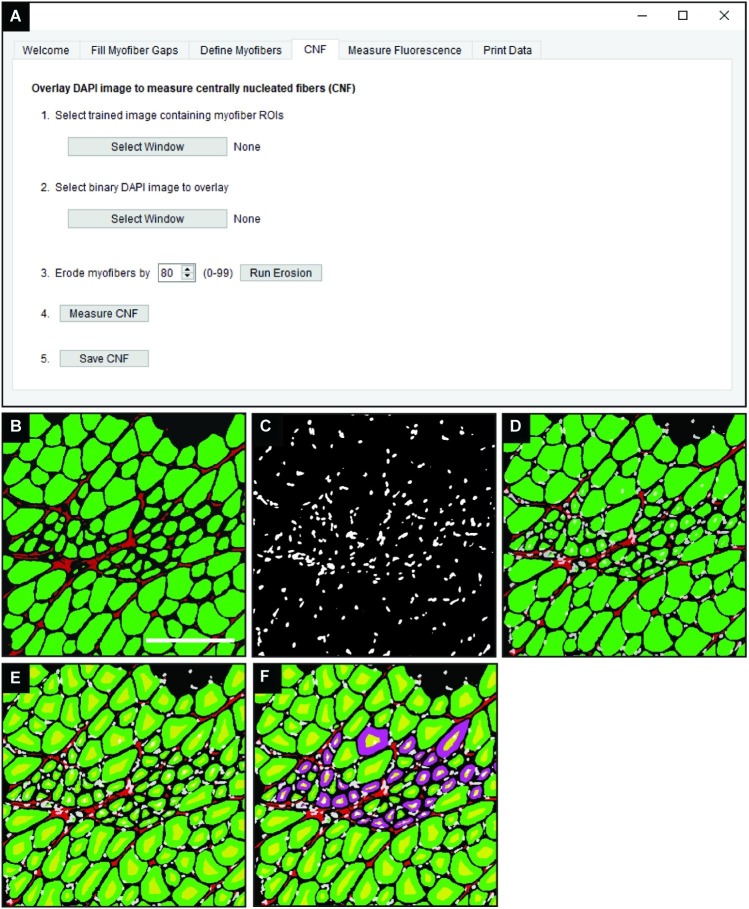
Detection of CNFs. **(A)** The QuantiMus user interface that is utilized for the detection of CNFs. **(B)** Representative cross-section of 4-week-old mdx mouse quadriceps previously classified using the “Myofiber Detection” function. **(C)** The corresponding DAPI image of the cross-section in **(B)**. **(D)** The overlay of classified and DAPI images. **(E)** Eroded myofibers (yellow) generated during the “Centrally-Nucleated Fibers” function. **(F)** The “Centrally-Nucleated Fibers” function end-product provides an image with CNFs labeled purple. Scale bar = 200 μm.

In the “CNF” tab of QuantiMus (Figure 4A), a classified image from the “Myofiber Detection” function (Figure 4B) is selected and a corresponding binarized DAPI-stained image of the same area (Figure 4C) is overlaid (Figure 4D). Using functions from the scikit-image toolbox (Van Der Walt et al., 2014), the area of myofibers (green ROIs) are eroded (Figure 4E, yellow regions) to exclude peripheral nuclei during the classification of CNFs. The degree of erosion is determined by a user-defined value from 0 to 99, where 99 is equal to approximately 99% myofiber erosion; 80 is used as the default (Figure 4A). If there is colocalization of the eroded myofiber ROI (Figure 4E, yellow regions) and DAPI signal, then a myofiber is classified as centrally nucleated. CNFs are then recolored to purple (Figure 4F), and myofiber CNF status is saved for later data export.

### Measuring Myofiber Fluorescence

**Figure 5:**
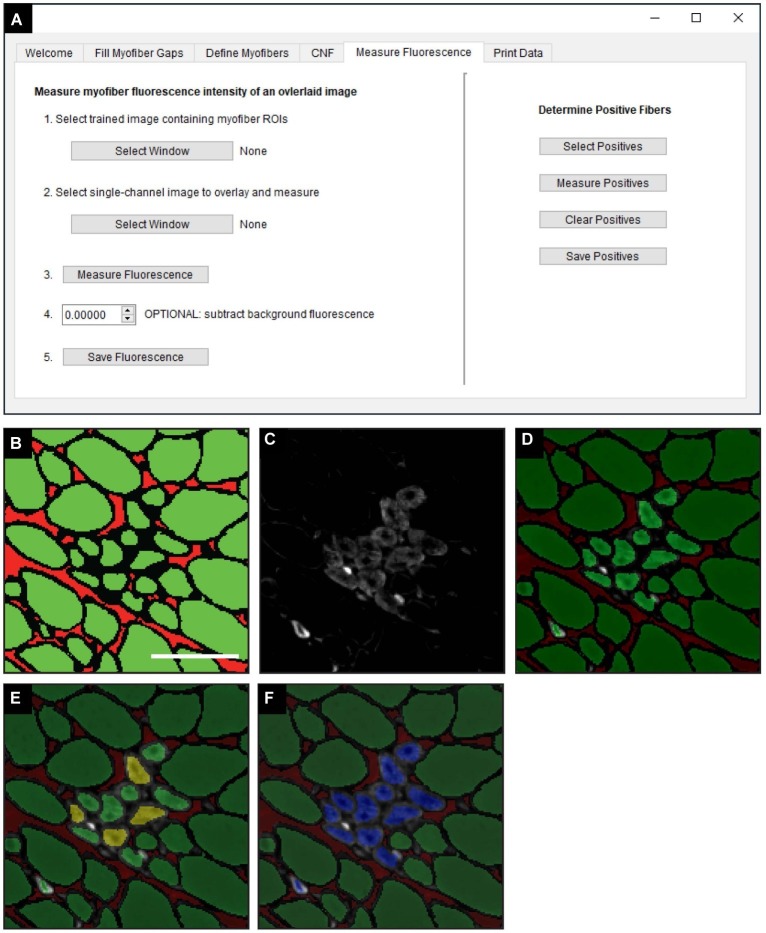
Measurement of fluorescence intensity in single myofibers. **(A)** QuantiMus user interface that is utilized for measuring the myofiber mean fluorescence intensity (MFI) of an overlaid fluorescence image. **(B)** Classified image generated by the “Myofiber Detection” function. **(C)** Fluorescence image of anti-eMyHC antibody-stained quadriceps. **(D)** Image in **(C)** is overlaid onto the corresponding classified image in **(B)**. **(E)** User-defined eMyHC^+^ myofibers (yellow) are used as a threshold for the automated determination of remaining eMyHC^+^ myofibers. **(F)** eMyHC^+^ myofibers are relabeled blue following the “Determine Positive Fibers” step. Scale bar = 100 μm.

The “Measure Fluorescence” function, selected in the “Measure Fluorescence” tab (Figure 5A), was used to measure the frequency of regenerating myofibers based on their expression of eMyHC, a marker of regeneration (Schiaffino et al., 1986). Classified images (Figure 5B) are superimposed with fluorescently labeled eMyHC images (Figure 5C) to yield an overlay (Figure 5D). Scikit-image functions are used to measure the NCAM and eMyHC mean fluorescence intensity (MFI), defined as the average fluorescence intensity of all pixels within a given myofiber (Van Der Walt et al., 2014). The “Determine Positive Fibers” feature (Figure 5A) is used to manually select a subset of myofibers in the overlay image (Figure 5D) with the lowest positive fluorescence signal (Figure 5E, yellow myofibers). These user-defined positive myofibers serve as a threshold to classify eMyHC positive myofibers in the entire cross-section; myofibers with an MFI equal to or greater than the threshold value are considered positive. After the algorithm completes the classification, positive myofibers are relabeled blue (Figure 5F). The MFI of all myofibers and the classification status (i.e., positive or not) is then saved for downstream analysis and data export.

### Data Export

**Figure 6:**
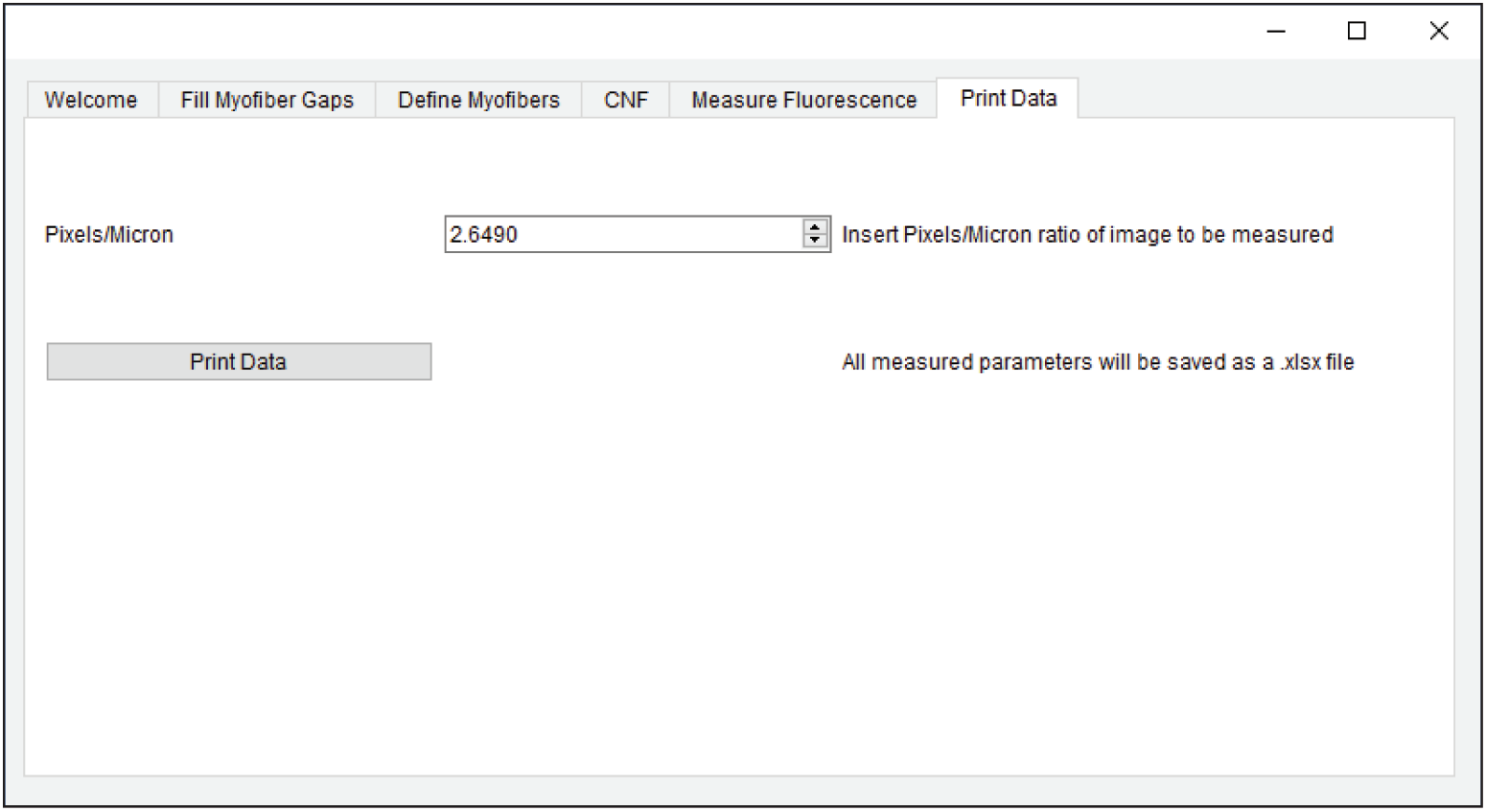
Data export. QuantiMus user interface utilized for the export of saved data.

All measurements generated in earlier steps are saved for final data export. When applicable, the pixel/micron ratio is entered in the “Print Data” tab of QuantiMus (Figure 6). It is important to note that when analyzing stitched images, pixel/micron ratios are distinct for each image and should be recorded during acquisition. In the BZ-X Analyzer software (Keyence) used to acquire images in this study, this value is found in the pop-up window that is displayed when inserting a scale bar. Alternatively, the “Set Scale”’ function in FIJI can be used to determine the pixel/micron ratio for images that contain scale bars inserted during acquisition. ROI number, myofiber area (μm^2^), minimum Feret diameter (μm), CNF classification, MFI, and regenerating myofiber classification are exported as an Excel (XLSX) file by clicking on “Print Data.”

## Results

### Image Processing Time Using Multiple Methods

Assessing morphometric features of entire muscle cross-sections removes potential biases inherent to random-sampling techniques and is recommended by the TREAT-NMD (Nagaraju and Willmann, 2009). The manual assessment of an entire muscle cross-section is time consuming, and the requirement for high computational processing power by some analysis software makes these tools inaccessible to some users (Kostrominova et al., 2013; Wen et al., 2017). QuantiMus was developed for the accurate evaluation of entire muscle cross-sections without requiring large computational resources. A laptop computer containing 12.0 gigabytes of random access memory and a 2.50 gigahertz critical processing unit (i7-6500U) was used to evaluate the processing time of an entire muscle cross-section using FIJI, SMASH, MyoVision or QuantiMus. 108 TIFF images of 4-week-old mdx mouse quadriceps taken with a 20x objective (1920 × 1440 pixels each) were stitched together with BZ-X Analyzer software to reconstruct the entire muscle cross-section. Our analysis showed that SMASH and QuantiMus had similar processing times, but QuantiMus classified myofibers and measured size with higher precision (Table 2). Using TIFF images, MyoVision was unable to complete image segmentation and myofiber measurement within a four-hour analysis time; this software could not process the image beyond the “Separated Seeds Step 10” step. It is important to note that MyoVision was able to processes a lower resolution PNG image of a full cross-section, however this analysis resulted in inaccurate myofiber detection and CSA measurements (data not shown). Together our analysis shows QuantiMus’ ability to rapidly assess morphology in a high content image of an entire muscle section.

**Table 2:** Comparison of image processing times using different methods.^*^

Method	Time (min)^a^	Fibers/cross section	CSA (μm^2^)^b^	Min Feret (μm)^c^
FIJI	133 ± 21	6,699 ± 153	745.53 ± 12.69	24.49 ± 0.88
QuantiMus	7 ± 1	7,638 ± 5	710.73 ± 0.38	23.10 ± 0.00
SMASH	5 ± 0.5	8,630 ± 0	813.86 ± 0.00	26.51 ± 0.00
MyoVision	Failed^d^	N.D.	N.D.	N.D.

*N.D., not determined*.

* *Data are the average ± standard error of the mean of three independent analyses of one entire muscle cross-section*.

a *Processing time (min) required to analyze myofiber size in full quadriceps cross-section image*.

b *Average cross-sectional area (CSA)*.

c *Average minimum Feret diameter*.

d *Analysis did not complete within a 4-h period*.

### Accuracy of Myofiber Classification and Cross-Sectional Area Measurement

**Figure 7:**
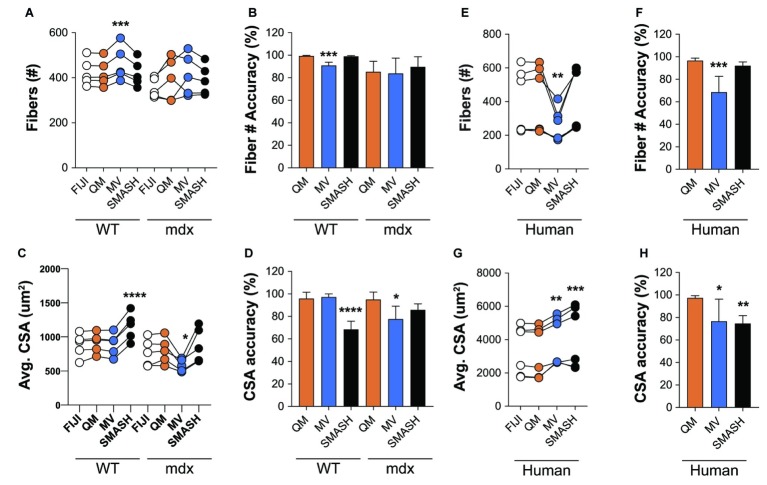
QuantiMus accurately measures myofiber CSA and minimum Feret diameter. **(A)** The number of myofibers detected using FIJI, QuantiMus (QM), MyoVision (MV), and SMASH in 4-week-old WT and mdx quadriceps. **(B)** The percent accuracy of the number of myofibers detected by each method. **(C)** The average (Avg) CSA (μm^2^) of myofibers in **(A)**. **(D)** The percent accuracy of average myofiber CSA for each method. Greater than 2,000 fibers from five representative fields, taken from two mice were used for each group. **(E)** The number of myofibers detected in human muscle. **(F)** The percent accuracy of myofiber classification for each method in **(E)**. **(G)** The Avg CSA (μm^2^) detected by each method in **(E)**. **(H)** The percent accuracy of average myofiber CSA for each method in **(G)**. Over 2,400 myofibers from six representative fields, taken from two patients were measured. Connected data points are indicative of a single image analyzed by each method. ^*^p < 0.05, ^**^p < 0.01, ^***^p < 0.001, and ^****^p < 0.0001 using a two-way repeated measures ANOVA with a multiple comparison test (main column effect). Statistics are compared to FIJI **(A,C,E,G)** or QM **(B,D,F,H)**.

We next tested the accuracy of QuantiMus in classifying myofibers and measuring CSA. QuantiMus was compared to SMASH, MyoVision and manual detection with FIJI, which was used to establish the ground truth. Due to the inability of MyoVision to completely process a stitched TIFF image of the entire muscle cross-section, we used a single image of a representative field taken at 20X for further analysis. We found that all methods were accurate and detected a similar number of myofibers, although MyoVision overestimated the number of myofibers in WT mice (Figures 7A,B). We also found that QuantiMus, MyoVision, and FIJI performed with similar accuracy when measuring the average myofiber CSA in WT mice but was overestimated by SMASH (Figures 7C,D). This observation is likely due to SMASH’s method of image segmentation where measured myofibers include some of the extracellular region leading to larger CSAs for each fiber (Smith and Barton, 2014). In mdx quadriceps, our analysis showed that the average size of myofibers defined by QuantiMus was similar to those measured with FIJI, but MyoVision grossly underestimated myofiber CSA (Figure 7C). We anticipate this finding to be a result of more ROIs of smaller size being inappropriately classified as true myofibers. As a result of over and underestimated CSA measurements, MyoVision and SMASH accuracies are variably influenced by different muscle conditions (i.e., healthy versus diseased muscle, Figure 7D). Taken together, although each program detected a similar number of myofibers, QuantiMus most accurately measured area across different muscle conditions.

We also assessed the ability of QuantiMus to accurately detect myofibers and measure myofiber CSA in de-identified tissue sections of archived human skeletal muscle. Biopsies were collected from patients with a suspected inflammatory myopathy, which after pathological assessment revealed no skeletal muscle involvement. We found that QuantiMus detected a similar number of myofibers compared to FIJI (Figure 7E) and was more accurate than MyoVision (Figure 7F), which detected fewer fibers (Figure 7E). We also evaluated CSA in cross-sections of human muscle. Although QuantiMus measured a similar average CSA compared to manual measurement using FIJI, SMASH and MyoVision measured an artificially larger CSA, thus reducing their accuracy (Figures 7G,H). Collectively, these results validate QuantiMus as an accurate and reliable tool for the rapid and accurate assessment of CSA in mouse and human skeletal muscle.

### Accuracy of Centrally Nucleated Fiber Classification and Cross-Sectional Area

**Figure 8:**
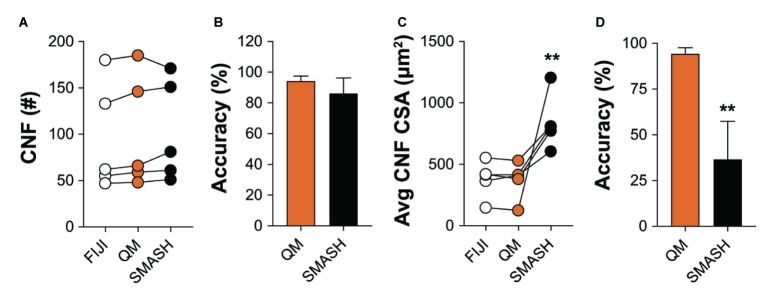
Defining CNFs. **(A)** The number of CNFs detected using manual measurement (FIJI), QuantiMus (QM), and SMASH in 4-week-old mdx quadriceps. **(B)** The percent accuracy of QuantiMus and SMASH to detect CNFs in **(A)**. **(C)** The average CSA (μm^2^) of CNFs detected by manual measurement (FIJI), QuantiMus, or SMASH. **(D)** The percent accuracy of QuantiMus and SMASH in measuring the CSA of detected CNFs compared to FIJI. Connected data points are indicative of a single image analyzed by each method. Five representative fields taken from two mice were used for analysis. ^**^p < 0.01 using a two-way repeated measures ANOVA with a multiple comparison test (main column effect) **(A,C)** or an paired two-tailed *t*-test **(B,D)**. Statistics are compared to FIJI **(A,C)** or QM **(B,D)**.

We also compared the ability of QuantiMus and SMASH to accurately determine and measure CNFs. MyoVision was not included in the comparison, as this analytical feature is not available in its current version. All methods measured CNF number in 4-week-old mdx quadriceps with similar accuracy (Figures 8A,B). We next determined the lower threshold of CNF size detection. Our analysis showed that the lowest CSA determined by FIJI and QuantiMus was on average 51.16 ± 8.61 and 50.32 ± 7.47 μm^2^, respectively. SMASH was unable to define CNFs smaller than on average 258.09 ± 22.48 μm^2^, likely explaining the overestimation of CSA (Figure 8C). Furthermore, this overestimation of CNF CSA by SMASH resulted in decreased accuracy (Figure 8D). The lower threshold of detection for CNF size led QuantiMus to reliably measure the CSA of CNFs (Figure 8D).

### Myofiber Typing of Full Muscle Cross-Sections

**Figure 9:**
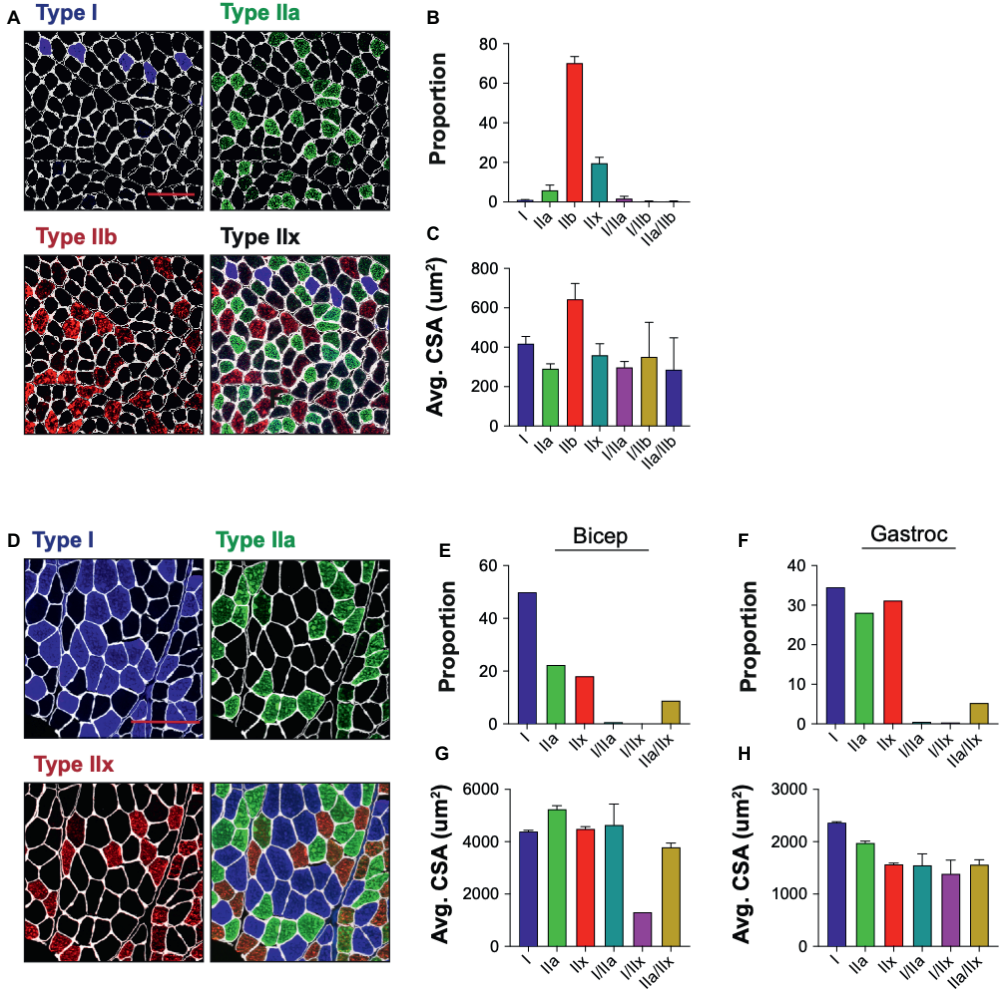
Myofiber typing of mouse and human muscle. **(A)** Representative image of WT mouse quadriceps cross-sections stained with antibodies against myosin heavy chain-specific isoforms. Blue = type I, green = type IIa, red = type IIb. Fibers with no isoform present are defined as type IIx. **(B)** The proportion of each myofiber type. **(C)** The average (Avg) cross-sectional area (CSA) of each fiber type. Data are displayed as the average ± SEM from full section measurements of four WT mice. A total of 25,757 fibers were measured. **(D)** Representative image of human cross-sections stained with antibodies against myosin heavy chain-specific isoforms. Blue = type I, green = type IIa, red = type IIx. **(E,F)** The proportion of each fiber type in human biceps brachii (Bicep) or gastrocnemius (Gastroc). (**G,H**) The Avg CSA of each fiber type in both muscle groups. Data are measured from full cross-sections and are displayed as the average ± SEM CSA of each patient sample **(G,H)**. 1,488 (Biceps) and 2,036 (Gastroc) fibers were measured. Scale bars = 100 **(A)** or 200 **(D)** μm.

Skeletal muscle is composed of multiple myofiber types that differ in their metabolic profiles and contractile properties, and can be classified based on their expression of specific myosin heavy chain isoforms. We tested the ability of QuantiMus to assess the myofiber expression and distribution of specific myosin heavy chain isoforms to define the proportion of type I, IIa, IIb, and IIx myofibers in entire cross-sections of WT mouse quadriceps (Figure 9A). As previously shown (Rederstorff et al., 2011), this analysis revealed that the majority of myofibers in quadriceps are type IIb fibers (70.3%), followed by type IIx (19.7%), type IIa (6.0%) and type I (1.1%) (Figure 9B). A small proportion of “hybrid” myofibers that express more than one myosin heavy chain isoform were also detected (Figure 9B; Matsuura et al., 2007). The quantification of myofiber type-specific CSA revealed that Type IIb is the largest in mouse quadriceps (Figure 9C). The accumulation of endogenous IgG in injured myofibers, despite blocking with commercially available mouse-on-mouse blocking reagents, precluded the ability to accurately type myofibers in mdx mice (data not shown). We also tested the ability of this tool to perform myofiber typing of various human muscles (Figure 9D). Our analysis showed the majority of myofibers in an entire muscle cross-section of biceps brachii from patient 1 were type I fibers (49.9%) followed by type IIa (22.4%) and IIx (18.2%) fibers (Figure 9E). The gastrocnemius of the second patient was primarily comprised of type I fibers (34.5%) followed by type IIx (31.2%) and IIa (28.1%) fibers (Figure 9F). Additionally, hybrid myofibers that expressed more than one myosin heavy chain isoform were also detected (Figures 9E,F). The average CSA of each myofiber type was also measured in the biceps (Figure 9G) and gastrocnemius (Figure 9H). Together, this analysis shows the capacity of QuantiMus to measure myofiber type distribution and their myofiber type-specific CSA in entire skeletal muscle cross-sections of mouse and human.

### Morphometric Analysis of Dystrophic Muscle Using QuantiMus

**Figure 10:**
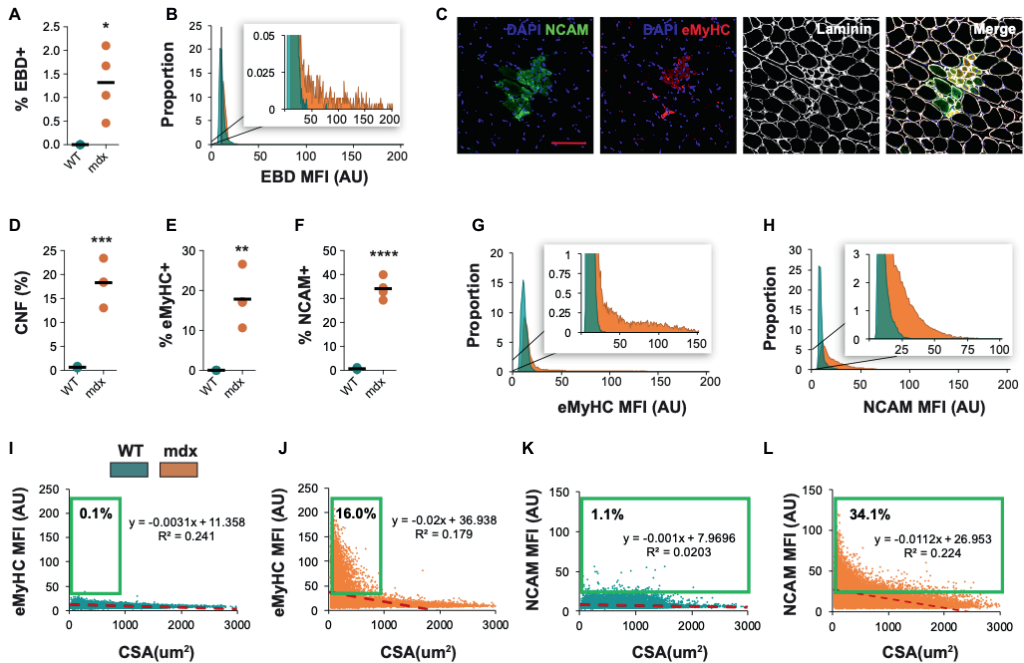
Morphometric analysis of dystrophic pathology in mdx mice. **(A)** Frequency of injured fibers (% EBD^+^) in entire quadriceps cross-sections of WT and mdx mice. **(B)** Histogram of muscle EBD expression showing individual myofiber EBD expression displayed as mean fluorescence intensity (MFI). *n* = 4 for each group. **(C)** Representative images of mdx mouse quadriceps cross-sections stained with DAPI (blue), NCAM (green), eMyHC (red), and laminin (white). The percentage of centrally-nucleated [CNF, **(D)**], eMyHC^+^ **(E),** and NCAM^+^ **(F)** fibers (of all fibers) in entire WT and mdx quadriceps cross-sections. **(G,H)** Histogram of eMyHC and NCAM expression showing individual myofiber expression displayed as MFI. Teal = WT, orange = mdx. **(I–L)** Linear regression analysis comparing eMyHC or NCAM MFI and myofiber CSA (μm^2^) in WT and mdx mice. Each dot represents a single myofiber. Red-dashed line corresponds to the equation generated by the linear regression analysis. *n* = 4 for each group. The boxed regions reflect data points that were above the background signal. Scale bar = 100 μm. AU = arbitrary units. Four-week-old mice were used. ^*^p < 0.05, ^**^p < 0.01, ^***^p < 0.001, and ^****^p < 0.0001 using an unpaired two-tailed *t*-test with Welch’s correction.

We also evaluated the ability of QuanitMus to assess the morphological features of dystrophic muscle, which contains more complex structural features (i.e., injured myofibers, varying myofiber size, and increased interstitial space). Evaluating myofiber injury provides a histological index of the severity of muscular dystrophy and is routinely evaluated by measuring the accumulation of Evans blue dye (EBD^+^) in injured myofibers (Straub et al., 1997; Hamer et al., 2002). We tested the ability of QuantiMus to measure muscle injury in EBD-injected WT and mdx mice by assessing the frequency of EBD^+^ fibers and their fluorescence intensity within the entire quadriceps cross-section. As shown previously (Straub et al., 1997), these studies revealed an increased proportion of injured fibers in mdx muscle compared to healthy controls (Figure 10A). Furthermore, we measured the EBD fluorescence intensity of individual myofibers with the “Measure Fluorescence” function. Notably, we found a broad range in the fluorescence intensity of EBD^+^ myofibers, which was absent in WT muscle (Figure 10B, inset).

QuantiMus was also used to histologically assess regeneration in healthy and dystrophic mouse muscle (Figure 10C). Using CNFs as an indicator of regeneration, we found the proportion of regenerating myofibers was elevated in mdx quadriceps and nearly absent in WT controls (Figure 10D; Torres and Duchen, 1987; McDonald et al., 2015). Nascent myotubes and regenerating myofibers can also be distinguished by their expression of developmental genes like eMyHC (DiMario et al., 1991) or NCAM (Capkovic et al., 2008). We, therefore, used QuantiMus to quantify the frequency of eMyHC^+^ and NCAM^+^ regenerating myofibers. Our analysis showed that the proportion of both eMyHC^+^ and NCAM^+^ myofibers was elevated in dystrophic muscle (Figures 10E,F), consistent with the increased proportion of CNFs. Moreover, the individual myofiber MFI of eMyHC and NCAM was higher in mdx mice compared to WT, suggesting a relative increased expression of these markers in mdx quadriceps (Figures 10G,H). The use of mean or median fluorescence intensity as an indirect measure of protein expression is a common technique used in a variety of semi-quantitative and quantitative platforms to report relative changes in protein or gene expression (McCabe et al., 2005; Gonçalves et al., 2011; Cirak et al., 2012; Banks et al., 2014; Van Battum et al., 2014; Omairi et al., 2017; Guirado et al., 2018; Guiraud et al., 2019). We next evaluated the relationship between CSA and eMyHC (Figures 10I,J) or NCAM (Figures 10K,L) MFI and found no correlation in WT mice (Figures 10I,K). However, eMyHC and NCAM MFI and CSA were negatively correlated in mdx mice, with the smallest myofibers having the highest MFI for these markers (Figures 10J,L). We also noted that there was a larger proportion of large NCAM^+^ myofibers (>1,000 μm^2^) that did not express eMyHC (compare Figures 10L,J), suggesting that the loss of eMyHC precedes the loss of NCAM as regenerating myofiber differentiate and grow. This observation also likely accounts for the increased proportion of NCAM^+^ myofibers compared to eMyHC^+^ myofibers in mdx mice (compare Figures 10E,F). The ability of QuantiMus to simultaneously measure CSA and MFI provided a novel analytic approach using linear regression analysis to quantify small myofibers expressing high levels of NCAM or eMyHC in regenerating muscle (Figures 10I–L).

## Discussion

The histomorphological and molecular assessment of injured or diseased skeletal muscle historically required time-consuming, manual methods. More recently, the histological evaluation of muscle tissue has been significantly accelerated by the development of semi-automated tools (Kostrominova et al., 2013; Smith and Barton, 2014; Wen et al., 2017; Reyes-Fernandez et al., 2019). These methods are able to successfully measure uniform myofibers of healthy muscle. However, these methods are not readily adaptable to the highly variable terrain of diseased muscle. Here, we introduce QuantiMus, a machine learning-based software, that addresses this and accelerates the histological evaluation of skeletal muscle. Benchmark comparisons validate this tool for the high-throughput and semi-automated determination of CSA, CNFs, fluorescence intensity, and myofiber type of entire skeletal muscle cross-sections.

The implementation of unique segmentation and SVM-based classification algorithms advances current morphometric methods by enhancing the ability to define individual myofibers and assess muscle pathology of entire muscle cross-sections. SVMs have been previously utilized to segment images (Wang et al., 2011, 2012) and classify objects (Adamiak et al., 2016) by using descriptors in a trained image as inputs for its supervised learning methods (Nayak et al., 2015). In the histological assessment of skeletal muscle, the SVM generates a complex nonlinear decision boundary between myofibers and other structural features, thereby removing the reliance on rigid user-defined parameters used by other methods that may lead to ROI misclassification. The novel development and implementation of the “Fill Myofiber Gaps” function also enhances accurate myofiber classification by “filling” artifactual gaps in the laminin-stained image used to define the myofiber perimeter. A binarized image with more precise boundaries is generated, consequently increasing the SVM’s ability to accurately classify ROIs as myofibers. An additional layer of accuracy has been added by implementing a semi-automated “Correction Filter” and a point-and-click user interface that allows investigators to manually change ROI status. These features allow the removal of myofibers at the edge of the muscle cross-section or in areas with poor sectional integrity. Further, the point-and-click feature allows the addition of true myofibers that were missed during classification. Together, these features integrate to form an accurate and user-friendly method for segmenting skeletal muscle images and classifying myofibers for downstream analysis.

We also implemented a novel myofiber eroding feature that facilitates the accurate classification of regenerating, CNFs ranging widely in size. We used an open-source algorithm (Pedregosa et al., 2012) to erode each myofiber by a percent area that scales with changing CSA size. In contrast, the use of hard-set parameters prevents the accurate discernment of small CNFs. As a result, SMASH failed to detect CNFs smaller than 258.09 ± 22.48 μm^2^, which are prevalent in regenerating regions of dystrophic muscle (Torres and Duchen, 1987). We attributed the increased CNF detection accuracy of QuantiMus to the erosion method, which is not adversely affected by heterogenous myofiber populations. Currently, the difficulty to resolve single nuclei in areas of high cellular density and overlapping nuclei limit our tool to detecting centrally-located nuclei. These limitations especially arise in settings of muscle inflammation, were infiltrating immune cells juxtaposed with myofibers make it difficult to discern infiltrating nuclei from peripheral myofiber nuclei. However, the ability to quantify peripheral nuclei in healthy muscle that lack densely compacted nuclei has been successfully performed (Wen et al., 2017).

We coupled machine learning-based classification and fluorescence intensity measurement methods to evaluate muscle function by simultaneously assessing the morphology and molecular features of a myofiber. We took rigorous measures, including labeling all sections on the same day; fluorescence signals were not saturated during image acquisition; specificity of the antibodies was validated, i.e., we used biological samples –mdx vs. WT– known to have increased expression of eMyHC and NCAM; fluorescently-labeled sections were protected from light; measurement of fluorescence intensity was done on unaltered images, to preserve a proportional relationship between MFI and protein expression. Combining these functions provided the capability to ascertain a relationship between myofiber size and eMyHC or NCAM fluorescence intensity at single-myofiber resolution. This becomes a critical analytical quality given that eMyHC^+^ and NCAM^+^ regenerating myofibers represent a minor fraction of total myofibers. Thus, small, but physiologically impactful changes in size or protein expression in this population may be missed because of their low prevalence. Although previous studies characterized NCAM expression in regenerating muscle, they did not measure CSA (Illa et al., 1992; Dubois et al., 1994). Here, measuring CSA and NCAM expression permitted a linear regression analysis that revealed a negative correlation between NCAM expression and myofiber size, which was also true for eMyHC^+^ myofibers. The reduction of eMyHC and NCAM expression with increasing myofiber size in mdx mice is consistent with other studies showing that these markers of regeneration are down-regulated with myofiber differentiation and/or growth (Covault and Sanes, 1986; Dubois et al., 1994; Agbulut et al., 2003; Schiaffino et al., 2015). Further, QuantiMus reliably identified a subset of very small myofibers expressing high levels of eMyHC and NCAM protein (Figures 10I–L green box), likely representing nascent myotubes present in mdx muscle (Charlton et al., 2000). Our study demonstrates that QuantiMus measures protein expression over a high dynamic range and accurately classifies small regenerating myotubes, to assess muscle regeneration with unprecedented sensitivity and accuracy.

QuantiMus was designed to segment and classify images for myofiber determination and measuring their fluorescence intensity. This design does not allow QuantiMus to measure unsegmented areas, preventing the quantification of injured or fibrotic areas over the entire cross-section. However, the “Measure Fluorescence” feature can be used to quantify the frequency of necrotic myofibers and their uptake of Evans Blue dye by measuring the MFI (Straub et al., 1997; Hamer et al., 2002). Similar approaches have been used to measure muscle membrane injury following acute injury detected with procion orange (Tidball and Wehling-Henricks, 2007). As expected, QuantiMus revealed a greater than 15-fold increase in EBD^+^ injured myofibers in mdx mice compared to WT mice. Further, measuring the MFI of EBD of all myofibers in a cross-section revealed a broad range in the fluorescence intensity of EBD in dystrophic muscle. We attribute this broad distribution to an increase in the number and/or size of lesions in the sarcolemma of a single myofiber that causes a larger and variable influx and accumulation of EBD. Measuring the MFI of EBD consequently becomes useful to measure injury when the frequency of injured myofibers is not different between experimental conditions, but the number of lesions per myofiber or size is significantly altered.

QuantiMus is an open-source software plug-in written in the Python programming language and is available at no cost. Python has a large open-source community that actively maintains a rich set of software libraries and packages that can be used to customize the functionality of QuantiMus for investigator-specific needs. The algorithms designed for QuantiMus were written to rapidly process high-content images and must be launched through the computer terminal, which may require some guidance to operate. To circumvent this limitation for novice users, we have provided extensive instructions for installation and program start-up of QuantiMus at https://quantimus.github.io.

Through extensive benchmarking, we validated QuantiMus as a novel machine learning-based tool for quantitative skeletal muscle morphometry. QuantiMus quantified the frequency of centrally-nucleated, injured, and regenerating myofibers in whole cross-sections with high precision. Further, QuantiMus rapidly typed myofibers based on the expression of MyHC isoforms. The capability to simultaneously measure fluorescence intensity and cross-sectional area provided a novel analytical approach for quantifying myofiber injury and regeneration. In summary, QuantiMus operates with equal and for many parameters exceeds the performance of existing software in quantifying histological and molecular features of muscle disease in human and mouse.

## Data Availability Statement

The datasets generated for this study are available on request to the corresponding author.

## Ethics Statement

The studies involving human participants were reviewed and approved by the Institutional Review Board at the University of California, Irvine. The patients/participants provided their written informed consent to participate in this study. The animal study was reviewed and approved by the University of California Irvine Institutional Animal Care and Use Committees.

## Author Contributions

JK, KE, and SVi conceptualized and designed experiments. KE developed the “Fill Myofiber Gaps” and “Myofiber Detection” algorithms. JK and JG developed the “Centrally-Nucleated Fibers” algorithm. KE, JG, AM, and JK implemented and developed software. JK, AM, and RY tested and validated software and performed analysis. RA, RY, PP, RR, and SVe prepared histological samples. TM provided human samples. JK, KE, AM, TM, and SVi prepared and edited the manuscript. All authors read and approved the final manuscript.

### Conflict of Interest

The authors declare that the research was conducted in the absence of any commercial or financial relationships that could be construed as a potential conflict of interest.

## References

[ref1] Adamiak K. Duch P. Ślot K. (2016). Object classification using support vector machines with kernel-based data preprocessing. Image Process. Commun. 21, 45–53. 10.1515/ipc-2016-0015

[ref2] Agbulut O. Noirez P. Beaumont F. Butler-Browne G. (2003). Myosin heavy chain isoforms in postnatal muscle development of mice. Biol. cell 95, 399–406. 10.1016/S0248-4900(03)00087-X 14519557

[ref3] Artan Y. (2011). Interactive image segmentation using machine learning techniques. 2011 Can. Conf. Comput. Robot Vis. 264–269. 10.1109/CRV.2011.42

[ref4] Banks G. B. Combs A. C. Odom G. L. Bloch R. J. Chamberlain J. S. (2014). Muscle structure influences utrophin expression in mdx mice. PLoS Genet. 10:e1004431. 10.1371/journal.pgen.1004431, PMID: 24922526 PMC4055409

[ref5] Capkovic K. L. Stevenson S. Johnson M. C. Thelen J. J. Cornelison D. D. W. (2008). Neural cell adhesion molecule (NCAM) marks adult myogenic cells committed to differentiation. Exp. Cell Res. 314, 1553–1565. 10.1016/j.yexcr.2008.01.021, PMID: 18308302 PMC3461306

[ref6] Charlton C. A. Mohler W. A. Blau H. M. (2000). Neural cell adhesion molecule (NCAM) and myoblast fusion. Dev. Biol. 221, 112–119. 10.1006/dbio.2000.9654, PMID: 10772795

[ref7] Cirak S. Feng L. Anthony K. Arechavala-Gomeza V. Torelli S. Sewry C.. (2012). Restoration of the dystrophin-associated glycoprotein complex after exon skipping therapy in Duchenne muscular dystrophy. Mol. Ther. 20, 462–467. 10.1038/mt.2011.248, PMID: 22086232 PMC3277241

[ref8] Covault J. Sanes J. R. (1985). Neural cell adhesion molecule (N-CAM) accumulates in denervated and paralyzed skeletal muscles. Proc. Natl. Acad. Sci. USA 82, 4544–4548. 10.1073/pnas.82.13.4544 3892537 PMC391139

[ref9] Covault J. Sanes J. R. (1986). Distribution of N-CAM in synaptic and extrasynaptic portions of developing and adult skeletal muscle. J. Cell Biol. 102, 716–730. 10.1083/jcb.102.3.716, PMID: 3512581 PMC2114104

[ref10] DiMario J. X. Uzman A. Strohman R. C. (1991). Fiber regeneration is not persistent in dystrophic (mdx) mouse skeletal muscle. Dev. Biol. 148, 314–321. 10.1016/0012-1606(91)90340-9, PMID: 1936568

[ref12] Dubois C. Figarella-Branger D. Pastoret C. Rampini C. Karpati G. Rougon G. (1994). Expression of NCAM and its polysialylated isoforms during mdx mouse muscle regeneration and in vitro myogenesis. Neuromuscul. Disord. 4, 171–182. 10.1016/0960-8966(94)90018-3, PMID: 7919966

[ref13] Dumont N. A. Bentzinger C. F. Sincennes M.-C. Rudnicki M. A. (2015). Satellite cells and skeletal muscle regeneration. Compr. Physiol. 5, 1027–1059. 10.1002/cphy.c140068, PMID: 26140708

[ref14] Ellefsen K. L. Settle B. Parker I. Smith I. F. (2014). An algorithm for automated detection, localization and measurement of local calcium signals from camera-based imaging. Cell Calcium 56, 147–156. 10.1016/j.ceca.2014.06.003, PMID: 25047761 PMC4162823

[ref15] Gonçalves M. A. F. V. Janssen J. M. Nguyen Q. G. Athanasopoulos T. Hauschka S. D. Dickson G.. (2011). Transcription factor rational design improves directed differentiation of human mesenchymal stem cells into skeletal myocytes. Mol. Ther. 19, 1331–1341. 10.1038/mt.2010.308, PMID: 21266958 PMC3129549

[ref16] Guirado R. Carceller H. Castillo-Gómez E. Castrén E. Nacher J. (2018). Automated analysis of images for molecular quantification in immunohistochemistry. Heliyon 4:e00669. 10.1016/j.heliyon.2018.e00669, PMID: 30003163 PMC6039854

[ref17] Guiraud S. Edwards B. Squire S. E. Moir L. Berg A. Babbs A. (2019). Embryonic myosin is a regeneration marker to monitor utrophin-based therapies for DMD. Hum. Mol. Genet. 28, 307–319. 10.1093/hmg/ddy353 30304405 PMC6322073

[ref18] Hamer P. McGeachie J. Davies M. Grounds M. (2002). Evans blue dye as an in vivo marker of myofibre damage: optimising parameters for detecting initial myofibre membrane permeability. J. Anat. 200, 69–79. 10.1046/j.0021-8782.2001.00008.x, PMID: 11837252 PMC1570883

[ref19] Illa I. Leon-Monzon M. Dalakas M. (1992). Regenerating and denervated human muscle fibers and satellite cells express neural cell adhesion molecule recogrued by monoclonal antibodies to natural killer cells. Ann. Neurol. 31, 46–52. 10.1002/ana.410310109, PMID: 1371910

[ref20] Kostrominova T. Y. Reiner D. S. Haas R. H. Ingermanson R. McDonough P. M. (2013). Automated methods for the analysis of skeletal muscle fiber size and metabolic type. Int. Rev. Cell Mol. Biol. 306, 275–332. 24016528 10.1016/B978-0-12-407694-5.00007-9

[ref21] Liu F. Structures T. (2009). Automated image segmentation of haematoxylin and eosin stained skeletal muscle cross-sections. J. Microsc. 6, 247–253. 10.1111/j.1743-6109.2008.01122.x.Endothelial PMC4079908 24118017

[ref22] Matsuura T. Li Y. Giacobino J.-P. Fu F. H. Huard J. (2007). Skeletal muscle fiber type conversion during the repair of mouse soleus: potential implications for muscle healing after injury. J. Orthop. Res. 25, 1534–1540. 10.1002/jor.20451, PMID: 17593537

[ref23] McCabe A. Dolled-Filhart M. Camp R. L. Rimm D. L. (2005). Automated quantitative analysis (AQUA) of in situ protein expression, antibody concentration, and prognosis. JNCI J. Natl. Cancer Inst. 97, 1808–1815. 10.1093/jnci/dji427 16368942

[ref24] McDonald A. A. Hebert S. L. Kunz M. D. Ralles S. J. McLoon L. K. (2015). Disease course in mdx:utrophin+/− mice: comparison of three mouse models of Duchenne muscular dystrophy. Phys. Rep. 3, e12391–e12391. 10.14814/phy2.12391, PMID: 25921779 PMC4425985

[ref25] Nagaraju K. Willmann R. (2009). Developing standard procedures for murine and canine efficacy studies of DMD therapeutics. Neuromuscul. Disord. 6, 247–253. 10.1111/j.1743-6109.2008.01122.x.Endothelial PMC2766092 19560356

[ref26] Nayak J. Naik B. Behera H. S. (2015). A comprehensive survey on support vector machine in data mining tasks: applications & challenges. Int. J. Database Theory Appl. 8, 169–186. 10.14257/ijdta.2015.8.1.18

[ref27] Omairi S. Hau K.-L. Collin-Hooper H. Montanaro F. Goyenvalle A. Garcia L.. (2017). Link between MHC fiber type and restoration of dystrophin expression and key components of the DAPC by tricyclo-DNA-mediated exon skipping. Mol. Ther. Nucleic Acids 9, 409–418. 10.1016/j.omtn.2017.10.014, PMID: 29246319 PMC6114118

[ref28] Pedregosa F. Varoquaux G. Gramfort A. Michel V. Thirion B. Grisel O. (2012). Scikit-learn: machine learning in python. J. Mach. Learn. Res. 12, 2825–2830. 10.1007/s13398-014-0173-7.2

[ref29] Rederstorff M. Castets P. Arbogast S. Lainé J. Vassilopoulos S. Beuvin M.. (2011). Increased muscle stress-sensitivity induced by selenoprotein N inactivation in mouse: a mammalian model for SEPN1-related myopathy. PLoS One 6:e23094. 10.1371/journal.pone.0023094, PMID: 21858002 PMC3152547

[ref30] Reyes-Fernandez P. C. Periou B. Decrouy X. Relaix F. Authier F. J. (2019). Automated image-analysis method for the quantification of fiber morphometry and fiber type population in human skeletal muscle. Skelet. muscle 9:15. 10.1186/s13395-019-0200-7 31133066 PMC6537183

[ref31] Rochlin K. Yu S. Roy S. Baylies M. K. (2010). Myoblast fusion: when it takes more to make one. Dev. Biol. 341, 66–83. 10.1016/j.ydbio.2009.10.024, PMID: 19932206 PMC2854170

[ref32] Schiaffino S. Gorza L. Sartore S. Saggin L. Carli M. (1986). Embryonic myosin heavy chain as a differentiation marker of developing human skeletal muscle and rhabdomyosarcoma. A monoclonal antibody study. Exp. Cell Res. 163, 211–220. 10.1016/0014-4827(86)90574-4, PMID: 3002828

[ref33] Schiaffino S. Rossi A. C. Smerdu V. Leinwand L. A. Reggiani C. (2015). Developmental myosins: expression patterns and functional significance. Skelet. Muscle 5:22. 10.1186/s13395-015-0046-6 26180627 PMC4502549

[ref34] Smith L. R. Barton E. R. (2014). SMASH - semi-automatic muscle analysis using segmentation of histology: a MATLAB application. Skelet. Muscle 4:21. 10.1186/2044-5040-4-21, PMID: 25937889 PMC4417508

[ref35] Straub V. Rafael J. A. Chamberlain J. S. Campbell K. P. (1997). Animal models for muscular dystrophy show different patterns of sarcolemmal disruption. J. Cell Biol. 139, 375–385. 10.1083/jcb.139.2.375, PMID: 9334342 PMC2139791

[ref36] Tedesco F. S. Dellavalle A. Diaz-manera J. Messina G. Cossu G. (2010). Repairing skeletal muscle: regenerative potential of skeletal muscle stem cells. J. Clin. Invest. 120, 11–19. 10.1172/JCI40373, PMID: 20051632 PMC2798695

[ref37] Tidball J. G. Wehling-Henricks M. (2007). Macrophages promote muscle membrane repair and muscle fibre growth and regeneration during modified muscle loading in mice in vivo. J. Physiol. 578, 327–336. 10.1113/jphysiol.2006.118265, PMID: 17038433 PMC2075127

[ref38] Torres L. F. B. Duchen L. W. (1987). The mutant mdx: inherited myopathy in the mouse: morphological studies of nerves, muscles and end-plates. Brain 110, 269–299. 10.1093/brain/110.2.269, PMID: 3567525

[ref39] Van Battum E. Y. Gunput R. A. F. Lemstra S. Groen E. J. N. Yu K. L. Adolfs Y.. (2014). The intracellular redox protein MICAL-1 regulates the development of hippocampal mossy fibre connections. Nat. Commun. 5:4317. 10.1038/ncomms5317, PMID: 25007825

[ref40] Van Der Walt S. Schönberger J. L. Nunez-Iglesias J. Boulogne F. Warner J. D. Yager N.. (2014). Scikit-image: image processing in python. PeerJ 2:e453. 10.7717/peerj.453, PMID: 25024921 PMC4081273

[ref41] Wang X. Y. Wang T. Bu J. (2011). Color image segmentation using pixel wise support vector machine classification. Pattern Recogn. 44, 777–787. 10.1016/j.patcog.2010.08.008

[ref42] Wang X. Y. Zhang X. J. Yang H. Y. Bu J. (2012). A pixel-based color image segmentation using support vector machine and fuzzy C-means. Neural Netw. 33, 148–159. 10.1016/j.neunet.2012.04.012, PMID: 22647833

[ref43] Wen Y. Murach K. A. Vechetti I. J. Fry C. S. Vickery C. D. Peterson C. A. (2017). MyoVision: software for automated high-content analysis of skeletal muscle immunohistochemistry. J. Appl. Physiol. 124, 40–51. 10.1152/japplphysiol.00762.2017 28982947 PMC6048460

[ref44] Zaitoun N. M. Aqel M. J. (2015). Survey on image segmentation techniques. Procedia Comput. Sci. 65, 797–806. 10.1016/j.procs.2015.09.027

